# Case Report: Eosinophilic Esophagitis in a Patient With a Novel STAT1 Gain-of-Function Pathogenic Variant

**DOI:** 10.3389/fimmu.2022.801832

**Published:** 2022-01-20

**Authors:** Ori Scott, Nigel Sharfe, Harjit Dadi, Linda Vong, Jenny Garkaby, Laura Abrego Fuentes, Jessica Willett Pachul, Sandra Nelles, Amit Nahum, Chaim M. Roifman

**Affiliations:** ^1^ Division of Immunology and Allergy, Department of Paediatrics, Hospital for Sick Children and University of Toronto, Toronto, ON, Canada; ^2^ The Canadian Centre for Primary Immunodeficiency and The Jeffrey Modell Research Laboratory for the Diagnosis of Primary Immunodeficiency, The Hospital for Sick Children, Toronto, ON, Canada; ^3^ Department of Gastroenterology, Trillium Health Partners, Mississauga Hospital, Mississauga, ON, Canada; ^4^ Pediatrics Department A, Soroka University Medical Center, Beer Sheva, Israel; ^5^ The Primary Immunodeficiency Research Laboratory, Faculty of Health Sciences, Ben-Gurion University of the Negev, Beer-Sheva, Israel

**Keywords:** STAT1, heterozygous mutation, eosinophilic esophagitis, candidiasis, atopy, immune dysregulation

## Abstract

**Background:**

STAT1 gain-of-function (GOF) is a primary immune dysregulatory disorder marked by wide infectious predisposition (most notably chronic mucocutaneous Candidiasis), autoimmunity, vascular disease and malignant predisposition. While atopic features have been described in some STAT1 GOF patients, they are not considered a predominant feature of the disease. Additionally, while eosinophilic gastrointestinal infiltration has been reported in some cases, this has always been described in the context of pre-existing oropharyngeal and/or esophageal Candidiasis.

**Clinical cases:**

Herein, we report 3 members of a multi-generational family diagnosed with STAT1 GOF caused by a novel mutation in the N-terminal domain, c.194A>C (p.D65A). The proband presented initially with a long-standing history of treatment-refractory eosinophilic esophagitis (EoE) without preceding gastrointestinal tract fungal infections, and her mother was diagnosed with esophagitis as well.

**Conclusion:**

EoE has been previously associated with alterations to STAT6 and STAT3 signaling pathways. The current report expands the possible association between JAK/STAT-related disorders and EoE, suggesting that EoE could be a primary disease manifestation of STAT1 GOF, even in the absence of oropharyngeal and/or esophageal Candidiasis.

## Introduction

Signal Transducer and Activator of Transcription (STAT) is a family of 7 structurally homologous transcription factors, with diverse functions both within and outside of the immune system. The canonical activation of STAT proteins follows a common sequence whereby extracellular receptor ligation prompts recruitment and cross-phosphorylation of tyrosine kinases of the Janus Kinase (JAK) family. This, in turn, creates a docking site for STAT proteins which are phosphorylated by JAK and undergo oligomerization to form active transcriptional complexes (1, 2). Within the STAT family, STAT1 plays a pivotal role in the signaling of various cytokines, most notably interferons and IL-27 (1–4). Over the past 15 years, monogenic disorders in the STAT1 gene have been described including biallelic loss of function (LOF) causing a profound combined immunodeficiency (CID) (5–7), a dominant negative LOF resulting in Mendelian Susceptibility to Mycobacterial Disease (MSMD) (8, 9), and an autosomal dominant gain-of-function (GOF) (10, 11). To date, over 400 cases of STAT1 GOF have been reported, making it the most common genetic cause identified in patients with chronic mucocutaneous Candidiasis (CMCC) (12, 13).

Since the initial description of STAT1 GOF as a syndrome of CMCC and hypothyroidism, its phenotypic spectrum has expanded dramatically to involve CID, intracranial vascular disease, various autoimmune manifestations, and malignant predisposition (10–17). Atopic manifestations have also been reported in STAT1 GOF patients, with a recent systematic review estimating 13.6% of patients to be affected by such features (13). Atopic presentations reported to date have included eczema, asthma and food allergies (12, 15, 18–21). Despite the above, atopy and eosinophilic disorders are still not commonly considered a predominant feature of STAT1 GOF, as opposed to other disorders affecting JAK/STAT signaling such as STAT3 LOF, STAT5B LOF or GOF, and JAK1 GOF (22). While gastrointestinal eosinophilia has been reported in a few STAT1 GOF patients (15, 23), this was typically identified following diagnosis of oropharyngeal/esophageal Candidiasis, and may have represented a secondary tissue reaction to fungal infection. Herein, we report a family of 3 generations affected by STAT1 GOF, with eosinophilic esophagitis (EoE) in the proband, and with esophagitis diagnosed in her mother as well.

## Methods

### Patient and Blood Samples

Data compiled prospectively and retrospectively from medical records were entered into the Canadian Centre for Primary Immunodeficiency Registry and Tissue Bank, which has been approved by the SickKids Research Ethics Board (protocol no. 1000005598). All patients provided written informed consent.

### T-Cell Proliferative Responses

Lymphocyte proliferative responses to mitogens, including PHA, anti-CD3 and anti-CD28 antibodies, were determined by thymidine incorporation, as reported previously (24). All assays were performed in triplicate and were compared with random normal controls.

### Genetic Diagnosis and Sequencing Confirmation

Genomic DNA was isolated from patient peripheral blood leukocytes using the Geneaid genomic DNA extraction kit (Geneaid Mini Kit; Sensi Capital Corp, Toronto, Ontario, Canada). Patient mutations were diagnosed *via* a clinical panel (Inborn Errors of Immunity Panel, Prevention Genetics), and confirmed by STAT1 Sanger sequencing using DTCS Quick Kit on an automated sequencer (Beckman-Coulter CEQ 8000).

### Cell Culture and Immunoblotting

STAT1-deficient U3A cells were obtained from ATCC. pCMV6-STAT1 was from OriGene. The STAT1 D65A mutant was created using QuikChange II XL site-directed mutagenesis (Agilent) and confirmed by sequencing. U3A cells were transfected with cDNA for wild type *STAT1* or D65A STAT1 using Lipofectamine 3000 (ThermoFisher Scientific), according to the manufacturer’s instructions. After 24 hours, transfected cells were serum-starved for 3 hours prior to stimulation with 100 ng/mL IFN-γ or 4ng/ml IFN-α. Whole-cell lysates were prepared in RIPA buffer, and 10ug per sample were loaded and analyzed by immunoblotting. Anti-phospho-Stat1 (Tyr701; 7649), anti-Stat1 (9172) and anti-GAPDH (2118) were all purchased from Cell Signaling Technology (MA, USA). Immunoblotting experiments were done in triplicates. All blots derive from the same experiment and were processed in parallel.

### STAT1-Dependent Gene Expression Assessment

U3A cells transfected with wild type or D65A *STAT1* were stimulated for 8 hours with either IFN-γ or IFN-α as described above, and mRNA levels of *CXCL10* and *CXCL9* were determined by quantitative real-time PCR. Expression data were normalized to the levels of the house-keeping gene GAPDH and are presented as mean and standard deviation from a total of 3 experiments. Statistical analysis was performed using the unpaired Student’s t-test.

## Case Description

### Proband (P-I)

**Figure 1:**
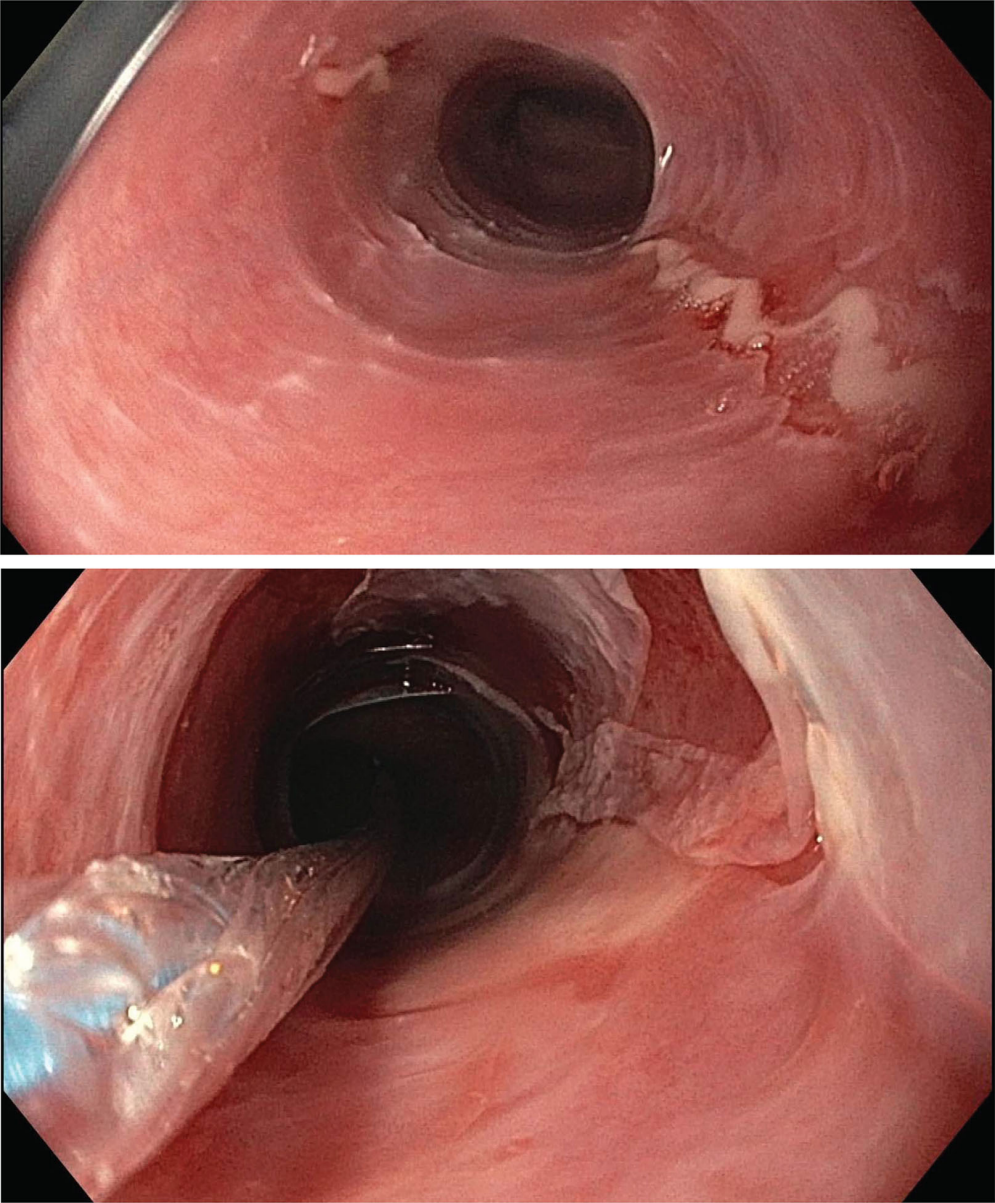
Endoscopic assessment of esophagitis in proband. Upper endoscopic assessment of the esophagus in our proband given history of recurrent esophageal strictures. Top: a ringed esophagus with linear ulcerations. Bottom: post balloon dilation, demonstrating sloughed, paper-thin esophageal mucosa without any evidence of deeper tears.

A 39-year-old female presented to our clinic for further work-up in the context of prolonged and treatment-refractory EoE. The patient first developed symptoms of dysphagia to solids in her late adolescence, but was not formally evaluated until the age of 31 when the dysphagia progressed to involve both solids and liquids, and following multiple episodes of food bolus impaction. Two initial upper endoscopies revealed a macroscopically abnormal esophagus with multiple strictures and rings (**Figure 1**); however, no eosinophilia was noted at the time, and no organisms were identified. Testing for H. pylori was negative. Special stains for acid-fast bacilli and fungal elements were negative. As the patient continued to suffer from symptoms highly suggestive of EoE requiring esophageal dilations every few months, she underwent a repeat endoscopy at the age of 34, this time demonstrating eosinophil-rich esophagitis (20-22 eosinophils per high-power field in both proximal and distal esophagus), with eosinophil clustering and degranulation, and again no organisms noted and negative fungal stain. She was formally diagnosed with EoE, which has been difficult to treat over the next 5 years. The patient was initially trialed on a six-food elimination diet, with no substantial symptomatic relief. She was subsequently treated with a regimen of inhaled fluticasone, montelukast, and a proton pump inhibitor (PPI), without success. The patient was finally switched to a regimen of budesonide slurry and a PPI, resulting in improvement in her esophageal eosinophilia, although she continues to require frequent dilations. Notably, during this period she developed one episode of Candida esophagitis, which was treated with Nystatin and has not recurred. This was her first and only life-time episode of esophageal Candidiasis, with no oropharyngeal thrush ever reported.

**Figure 2:**
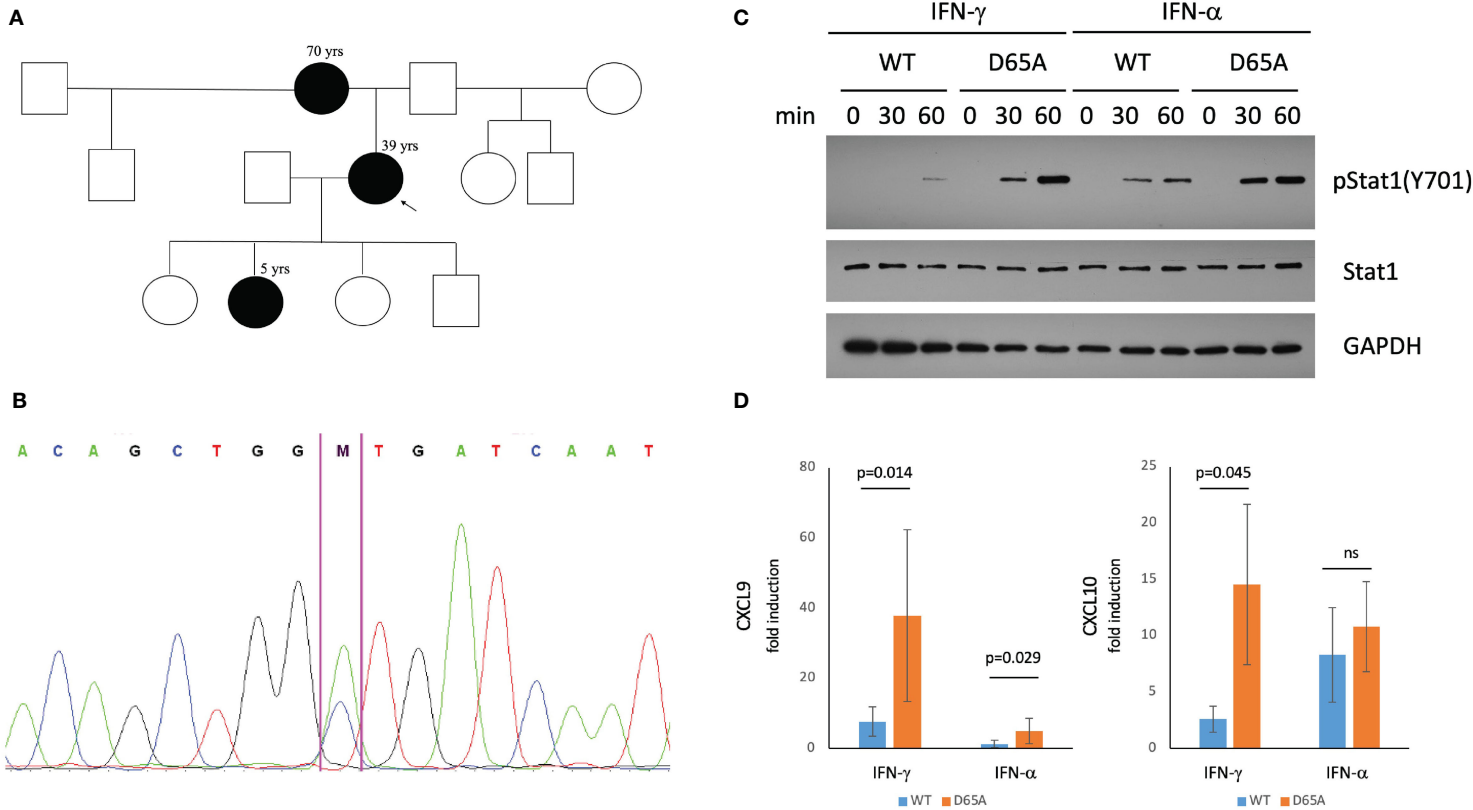
A *STAT1* mutation identified in our patients causes STAT1 gain-of-function. **(A)** Pedigree of a three-generational family affected by *STAT1* mutation (black). The proband (arrow) suffered from eosinophilic esophagitis and chronic mucocutaneous Candidiasis (CMCC). Her mother had a history of life-long esophagitis and CMCC. The proband’s daughter was affected by CMCC, recurrent acute otitis media, and atopic dermatitis. **(B)** Confirmatory sequencing of the *STAT1* gene in our patients revealed a heterozygous mutation, c.194A>C (p.D65A) in the N-terminal domain. **(C)** Immunoblotting of STAT1-null U3A cells transfected with either wildtype or D65A STAT1 demonstrated normal total levels of STAT1 in the mutant, with elevated levels of pSTAT1 (Tyr701) following stimulation with either IFN-γ or IFN-α. **(D)** Quantitative real-time PCR demonstrated increased fold induction of CXCL9 in D65A U3A cells after stimulation with either IFN-γ or IFN-α, with increased fold induction of CXCL10 following stimulation with IFN-γ. ns, not significant.

On our review of the patient’s infectious history, she endorsed a long-standing history of vaginal Candidiasis in her adult life, occurring several times per year. The infections typically responded to over-the-counter treatment with miconazole or clotrimazole, but recurred shortly after treatment. She also reported on previous fungal scalp infections, requiring the use of a medicated coal tar/chloroxylenol-based shampoo. She denied any other fungal infections, and had no history of viral, bacterial, Mycobacterial or opportunistic infections. She had no autoimmune, endocrine, autoinflammatory or malignant disease, and no other atopic features. She had received all age-appropriate vaccinations without difficulty. Review of the patient’s family history revealed that her mother (further described below) had a history of CMCC, and had recently been diagnosed with chronic esophagitis requiring dilations. The patient’s father had type 2 diabetes mellitus, hypertension, and coronary artery disease. Parents were both Canadian of mixed English-Irish ancestry, and non-consanguineous. The patient’s 3 half-siblings (1 maternal half-brother and 2 paternal half-siblings) were all healthy. One of the patient’s daughters (5 years old; further described below) was noted to have CMCC, recurrent acute otitis media (AOM) and atopic dermatitis. The patient’s three other children (ages 4-17 years) were healthy. A family pedigree is outlined in **Figure 2A**.

An immune evaluation of the proband (detailed in **Table 1**) was normal, including complete blood count and differential (CBC-Diff), lymphocyte subset analysis, total immunoglobulins, vaccine-specific antibody titres, and T-cell mitogen stimulation responses.

**Table 1:** Immune laboratory values for patients.

	P-I	P-II	P-III
**White blood cells**	5.12 x10ˆ9/L (4.37 - 9.68 x10ˆ9/L)	4.4 x10ˆ9/L (4.37 - 9.68 x10ˆ9/L)	5.79 x10ˆ9/L (4.23 - 9.99 x10ˆ9/L)
**Hemoglobin**	133 g/L (106 - 135 g/L)	131 g/L (106 - 135 g/L)	135 g/L (112 - 141 g/L)
**Platelets**	352 x10ˆ9/L (186 - 353 x10ˆ9/L)	271 x10ˆ9/L (186 - 353 x10ˆ9/L)	334 x10ˆ9/L (203-431 x10ˆ9/L)
**Neutrophils**	3.38 x10ˆ9/L (1.45 - 6.75 x10ˆ9/L)	2.83 x10ˆ9/L (1.45 - 6.75 x10ˆ9/L)	1.88 x10ˆ9/L (1.45 - 6.75 x10ˆ9/L)
**Lymphocytes**	1.17 x 10ˆ9/L (1.16 - 3.18 x 10ˆ9/L)	**0.98 x 10ˆ9/L** (1.16 - 3.18 x 10ˆ9/L)	3.46 x 10ˆ9/L (1.34 – 4.12 x 10ˆ9/L)
**Monocytes**	0.40 x 10ˆ9/L (0.29 - 0.71 x 10ˆ9/L)	0.45 x 10ˆ9/L (0.29 - 0.71 x 10ˆ9/L)	0.29 x 10ˆ9/L (0.27 – 0.81 x 10ˆ9/L)
**Eosinophils**	0.11 x 10ˆ9/L (0.03 - 0.27 x 10ˆ9/L)	0.04 x 10ˆ9/L (0.03 - 0.27 x 10ˆ9/L)	0.11 x 10ˆ9/L (0.06 - 0.97 x 10ˆ9/L)
**Basophils**	0.05 x 10ˆ9/L (0.01 - 0.05 x 10ˆ9/L)	0.06 x 10ˆ9/L (0.01 - 0.05 x 10ˆ9/L)	0.04 x 10ˆ9/L (0.01 - 0.06 x 10ˆ9/L)
**CD3+**	876 cells/uL (700-2100 cells/uL)	876 cells/uL (700-2100 cells/uL)	2220 cells/uL (1578-3707 cells/uL)
**CD3+CD8+**	269 cells/uL (200-900 cells/uL)	230 cells/uL (200-900 cells/uL)	733 cells/uL (472-1107 cells/uL)
**CD3+CD4+**	520 cells/uL (300-1400 cells/uL)	510 cells/uL (300-1400 cells/uL)	1219 cells/uL (870-2144 cells/uL)
**CD16+CD56+**	121 cells/uL (90-600 cells/uL)	**60 cells/uL** (90-600 cells/uL)	**153 cells/uL** (155-565 cells/uL)
**CD19+**	173 cells/uL (100-500 cells/uL)	131 cells/uL (100-500 cells/uL)	862 cells/uL (434-1274 cells/uL)
**CD4/CD8 Ratio**	1.94 (0.9-3.4)	2.2 (0.9-3.4)	1.66 (1.26-2.9)
**Total protein**	77 g/L (63 - 82 g/L)	67 g/L (63 - 82 g/L)	69 g/L (62 - 77 g/L)
**Albumin**	46 g/L (35 - 50 g/L)	41 g/L (35 - 50 g/L)	44 g/L (35 - 47 g/L)
**IgA**	3.3 g/L (0.7-4.2 g/L)	1.0 g/L (0.7-4.2 g/L)	0.9 g/L (0.3-1.5 g/L)
**IgG**	11.4 g/L (5.5-16.3 g/L)	8.3 g/L (5.5-16.3 g/L)	8.4 g/L (5.4-13.6 g/L)
**IgM**	0.8 g/L (0.3-2.9 g/L)	1.4 g/L (0.3-2.9 g/L)	1.1 g/L (0.5-1.9 g/L)
**Diphtheria toxoid IgG**	0.48 IU/mL (>0.1 IU/mL)	N/D	0.70 IU/mL (>0.1 IU/mL)
**Tetanus toxoid IgG**	0.34 IU/mL (>0.1 IU/mL)	0.36 IU/mL (>0.1 IU/mL)	0.46 IU/mL (>0.1 IU/mL)
**Measles serology**	Non-reactive	Non-reactive	Reactive
**Mumps serology**	Reactive	Reactive	Reactive
**Rubella serology**	Reactive	Reactive	Reactive
**Varicella serology**	N/D	Reactive	N/D
**T-cell PHA stimulation index**	1029 (>300)	709 (>300)	**135 (>300)**

N/D, not done. Bolded values are outside of normal reference range.

### Proband’s Mother (P-II)

This 70-year-old woman reported on a 50-year history of dysphagia and choking episodes, but was only evaluated by a gastroenterologist for the first time at the age of 66. Endoscopic assessment showed a severely narrowed esophagus, with biopsy demonstrating extensive tissue fibrosis, rare eosinophils in the proximal esophagus, and no organisms identified. At that time, there was evidence of marked basal cell hyperplasia, which 3 years later progressed to a squamous cell papilloma, currently managed conservatively. On review of infectious history, the patient had suffered from CMCC since the age of 5 years. In the first decade of life this included episodes of oral thrush, whereas in adulthood she suffered from recurrent skin fungal infections, with no oropharyngeal, vaginal or nail infections noted. She never had any autoimmune or endocrine concerns. Laboratory evaluation in this patient was notable for lymphopenia, with reduced natural killer (NK) cell counts. Her evaluated B and T-cell responses were within normal range (**Table 1**).

### Proband’s Daughter (P-III)

This 5-year-old girl was affected by CMCC since early infancy, including persistent oral thrush in the first year of life, followed by recurrent fungal scalp infections throughout early childhood. She also had a history of recurrent AOM infections with subsequent hearing loss necessitating tympanostomy tube insertion, but no other sinopulmonary infections. This patient had no concerns for dysphagia or feeding difficulty. There was no history of autoimmunity or endocrinopathy. Her immune evaluation showed a borderline low NK cell count, as well as decreased T-cell proliferation in response to PHA stimulation (**Table 1**).

## Diagnostic Assessment

### Genetic Testing

All patients underwent a genetic evaluation *via* a clinical Inborn Errors of Immunity panel (PreventionGenetics), demonstrating a novel heterozygous missense variant in the N-terminal domain of STAT1: c.194A>C (p.D65A; **Figure 2B**). This variant has not been reported in large population databases or literature. Another missense mutation affecting the same amino acid residue, c.193G>A (p.D65N) was recently reported as causing STAT1 GOF (25).

### Functional Assessment

Immunoblotting assessment of U3A cells transfected with either wildtype or D65A STAT1 revealed comparable levels of total STAT1 between wildtype and D65A. While no pSTAT1 was seen at baseline following serum-starvation, stimulation with either IFN-γ or IFN-α resulted in a substantial increase in pSTAT1 in the mutant compared with wildtype, for both timepoints measures (30 and 60 minutes; **Figure 2C**), confirming a GOF phenotype. In regards to gene expression, we evaluated the induction of two known STAT1 targets, *CXCL9* and *CXCL10*, following stimulation with either IFN-γ or IFN-α. The fold induction of CXCL9 was significantly higher in D65A compared with wildtype cells, after either IFN-γ or IFN-α (*p=0.014* and *p=0.029*, respectively). Fold induction of CXCL10 was significantly increased in D65A after stimulation with IFN-γ (*p=0.045*) but not IFN-α (**Figure 2D**).

## Outcome and Management

Following their diagnosis of esophagitis, both the proband and her mother have continued to require frequent esophageal dilations, as response to other standard EoE therapies including swallowed steroids, PPI and elimination diet has been limited. The possibility of initiating treatment with a JAK inhibitor has been considered by our team; however, there has been no clear indication for initiation of such a therapy in this family. Specifically, no severe autoimmunity, lymphoproliferation or severe refractory infections have been reported by the family, whereas atopy or EoE have not been well-studied to date as indications for treatment with JAK inhibitors. The proband’s mother is also followed conservatively for esophageal squamous cell papilloma, as described above. The proband’s daughter currently requires no treatment.

## Discussion

This report presented a family affected by STAT1 GOF with severe, treatment-refractory EoE as the presenting manifestation in the proband. Esophagitis requiring multiple dilations was also diagnosed in the proband’s mother, while the proband’s daughter displayed eczema, but had not yet developed esophagitis at the age of 5.

The association between EoE and STAT proteins, in particular STAT6, was first described in 2003 given the role of STAT6 in IL-4 and IL-13 signaling, and the demonstration that STAT6 deficient mice were resistant to an experimental model of IL-13-induced EoE (26). A decade later, functional studies in esophageal-derived cell cultures from EoE patients showed that STAT6 was required for transcriptional activation of the eosinophil chemoattractant, eotaxin 3 (27), an important player in EoE pathophysiology (28). Additionally, STAT6 inhibition using JAK inhibitors such as Ruxolitinib or Leflunomide resulted in significant reduction in eotaxin 3 expression (29). From a genetic standpoint, a genome-wide association study identified a significant association of EoE with variants at the STAT6 locus (30). Moreover, a recent study of pediatric EoE patients revealed that patients harbouring certain STAT6 variants were at a higher risk of disease relapse after a year of PPI treatment, compared with children not carrying such variants (31).

While variants in STAT6 have been identified in otherwise healthy individuals with EoE, a connection between primary immunodeficiency disorders (PIDD) and EoE has also emerged in recent years. Reports of EoE in patients with common variable immunodeficiency (CVID) were initially published in 2016 (32), with one population database study showing a higher prevalence of both CVID and IgA deficiency among patients with EoE (33). In 2018, a survey of the United States Immunodeficiency Network (USIDNET) identified 63 patients with eosinophilic gastrointestinal disease (EGID), of whom 50 had EoE. Almost half of the patients identified had CVID, while others had diagnoses of autosomal dominant hyper IgE syndrome (AD-HIES), chronic granulomatous disease, or CID. However, approximately one third of the patients had oropharyngeal/esophageal Candidiasis, and it was unknown what proportion of those had developed EoE prior to the onset of EGID (34). Within the realm of STAT-related PIDD, a National Institute of Health (NIH) study evaluating gastrointestinal manifestations in patients with AD-HIES identified high rates of esophageal eosinophilic infiltration, with other abnormalities including food impaction, esophageal tortuosity, ulcerations, or strictures requiring dilations, suggestive of possible EoE in this patient population (35).

Given the above-described association between AD STAT3 LOF and esophageal disease/EoE, it stands to reason that STAT1 GOF might cause EoE given loss of STAT3 promoter binding activity in this disorder. Additionally, STAT1 itself may also be implicated in EoE, given its role in IL-5 signaling in eosinophils (36, 37). Work by Nguyen et al. examined esophageal and peripheral blood samples of untreated EoE patients, comparing them with treated EoE patients as well as healthy controls. They found that transcript levels of not only STAT6 but also STAT1 were elevated in esophageal tissue of untreated EoE patients compared with control. Additionally, peripheral eosinophils and T-cells both showed enhanced phosphorylation of both STAT1 and STAT6. The authors suggested that this may be in keeping with the current understanding of EoE as a “mixed-type” immunological disorder, involving both Th2 and non-Th2 components (38).

From a therapeutic standpoint, JAK-inhibitors have emerged in recent years as a non-curative option for STAT1 GOF patients, and a potential bridge to HSCT, which may alleviate some immune dysregulatory features (20, 39, 40). However, data on their efficacy with respect to atopic disease manifestations in STAT1 GOF is scarce. Furthermore, concerns have been raised regarding increased predisposition to opportunistic and invasive infections as a result of treatment with JAK-inhibitors (41). Therefore, further evidence may be required prior to initiating treatment with JAK-inhibitors for EoE in the context of STAT1 GOF.

In summary, the current report describes a novel mutation in the STAT1 N-terminal domain leading to a GOF phenomenon, and with a concurrent predominant presentation of eosinophilic esophagitis in the proband, as well as esophagitis in her mother. The main limitations of the current report are its single-family scope, and the possibility that EoE and STAT1 GOF are concurrently present in a non-causative manner. Indeed, it is conceivable that the proband and her mother both developed esophagitis which is STAT1-independent. Further reports identifying this association, as well as rigorous mechanistic studies, would be required to establish with certainty whether and how STAT1 GOF may cause EoE. We suggest maintaining a high index of suspicion for EoE in STAT1 GOF patients presenting with dysphagia or food impaction, even in the absence of oropharyngeal/esophageal Candidiasis. We further propose that STAT1 GOF be considered in the work-up of patients presenting with refractory or atypical EoE, especially when esophagitis is familial, and in the context of other features of STAT1 GOF such as CMCC.

## Data Availability Statement

The original contributions presented in the study are included in the article/supplementary material. Further inquiries can be directed to the corresponding author.

## Ethics Statement

The studies involving human participants were reviewed and approved by SickKids research ethics board (protocol 1000005598). Written informed consent to participate in this study was provided by the participants’ legal guardian/next of kin.

## Author Contributions

Patient clinical evaluation by OS, JG, LA, JW, SN, AN, and CR. Laboratory analysis performed by NS and HD. The first manuscript draft was written by OS. All authors critically revised and approved of the final version of the manuscript.

## Funding

This work was supported by Immunodeficiency Canada, The Canadian Centre for Primary Immunodeficiency and The Jeffrey Modell Research foundation (CMR). Salary support for OS has been provided by the Ontario Ministry of Health Clinician Investigator Program, the Hospital for Sick Children Clinician Scientist Training Program, the Canadian Child Health Clinician Scientist Training Program, and the Canadian Institutes of Health Research (CIHR) Doctoral Award: Frederick Banting and Charles Best Canada Graduate Scholarship.

## Conflict of Interest

The authors declare that the research was conducted in the absence of any commercial or financial relationships that could be construed as a potential conflict of interest.

## Publisher’s Note

All claims expressed in this article are solely those of the authors and do not necessarily represent those of their affiliated organizations, or those of the publisher, the editors and the reviewers. Any product that may be evaluated in this article, or claim that may be made by its manufacturer, is not guaranteed or endorsed by the publisher.
